# Improved cytometric analysis of untouched lung leukocytes by enzymatic liquefaction of sputum samples

**DOI:** 10.1186/s12575-022-00181-z

**Published:** 2022-11-17

**Authors:** Giulia Santopolo, Antonio Clemente, Estrella Rojo-Molinero, Sara Fernández, María Concepción Álvarez, Antonio Oliver, Roberto de la Rica

**Affiliations:** 1 Multidisciplinary Sepsis Group, Hospital Universitario Son Espases, Health Research Institute of Balearic Islands (IdISBa), 07120 Palma, Spain; 2 Department of Chemistry, University of the Balearic Islands, Palma, Spain; 3 Microbiology Department, Hospital Universitario Son Espases, Health Research Institute of Balearic Islands (IdISBa), Palma, Spain; 4 CIBER de Enfermedades Infecciosas (CIBERINFEC), Madrid, Spain

**Keywords:** Cytometry of sputum immune cells, Catalase, Enzymatic liquefaction of respiratory samples, Hydrogen peroxide, Dithiothreitol

## Abstract

**Background:**

Phenotyping sputum-resident leukocytes and evaluating their functional status are essential analyses for exploring the cellular basis of pathological processes in the lungs, and flow cytometry is widely recognized as the gold-standard technique to address them. However, sputum-resident leukocytes are found in respiratory samples which need to be liquefied prior to cytometric analysis. Traditional liquefying procedures involve the use of a reducing agent such as dithiothreitol (DTT) in temperature-controlled conditions, which does not homogenize respiratory samples efficiently and impairs cell viability and functionality.

**Methods:**

Here we propose an enzymatic method that rapidly liquefies samples by means of generating O_2_ bubbles with endogenous catalase. Sputum specimens from patients with suspected pulmonary infection were treated with DTT, the enzymatic method or PBS. We used turbidimetry to compare the liquefaction degree and cell counts were determined using a hemocytometer. Finally, we conducted a comparative flow cytometry study for evaluating frequencies of sputum-resident neutrophils, eosinophils and lymphocytes and their activation status after liquefaction.

**Results:**

Enzymatically treated samples were better liquefied than those treated with DTT or PBS, which resulted in a more accurate cytometric analysis. Frequencies of all cell subsets analyzed within liquefied samples were comparable between liquefaction methods. However, the gentle cell handling rendered by the enzymatic method improves cell viability and retains in vivo functional characteristics of sputum-resident leukocytes (with regard to HLA-DR, CD63 and CD11b expression).

**Conclusion:**

In conclusion, the proposed enzymatic liquefaction method improves the cytometric analysis of respiratory samples and leaves the cells widely untouched for properly addressing functional analysis of lung leukocytes.

**Supplementary Information:**

The online version contains supplementary material available at 10.1186/s12575-022-00181-z.

## Introduction

Immunophenotyping of sputum-resident leukocytes is a valuable approach for monitoring local immune responses in pulmonary infections [1–4] or allergic reactions [5], as well as airways diseases with autoinflammatory and/or autoimmune features [6–12]. The lung is a major immunological organ that harbors complex interactions between immune and structural cells which are essential to sustain the host homeostasis [13]. Thus, unraveling the cell types and functions that contribute to lung immunity has tremendous potential to better understand susceptibility to infections and aid in the definition of inflammatory stages in chronic airways diseases [14, 15]. These analyses require accurate identification and characterization of individual cell populations and the gold-standard technique to address them is flow cytometry. In spite of sputum samples being a valuable source of lung leukocytes from central airways (a region of the lung which is difficult to sample non-invasively) their cytometric analysis is hampered because they need to be liquefied prior to analysis. Moreover, sputum contains contaminating epithelial cells and variable amounts of debris which make the cytometric analysis challenging.

**Fig. 1:**
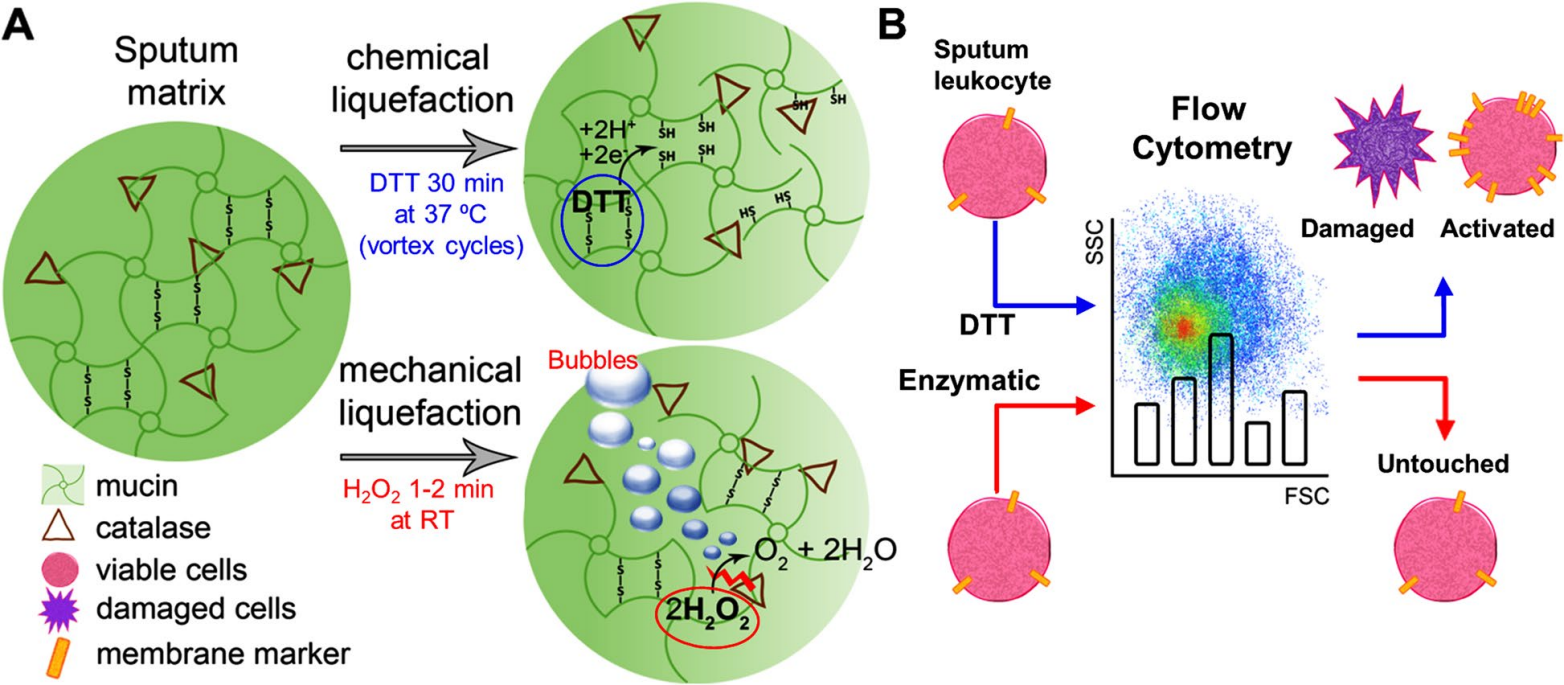
Schematic illustration of methods for liquefying respiratory samples and comparative cytometric analysis of sputum leukocytes. **A** DTT reduces disulfide bonds cross-linking mucins in the matrix, and the enzymatic method uses endogenous catalase from sputum samples for producing oxygen bubbles, which mechanically disrupts cross-linked mucins. **B** The enzymatic liquefaction of sputum samples improves the single-cell analysis of sputum-resident leukocytes by flow cytometry, ensures the cell viability and holds their activation status unaltered

Standard protocols for liquefying respiratory samples are based on reducing disulfide bonds cross-linking mucins in the matrix (Fig. 1A) [16, 17]. The most frequently used reducing agent is DTT, despite its negative impact on cell functionality and detection of surface markers [18, 19]. Using mild buffer treatments (e.g., PBS) can circumvent these issues [20], but they require intense mechanical dispersion and do not liquefy highly viscous sputum samples, which jeopardizes the proper handling of cells. Therefore, developing better liquefying methods that preserve the cell integrity is still a challenge in flow cytometry of sputum samples.

In this manuscript we introduce an alternative approach for sample liquefaction that improves the cytometric analysis of sputum-resident leukocytes. It consists of using endogenous catalase enzymes for producing oxygen bubbles that mechanically disrupt respiratory samples (Fig. 1A). Catalase is a ubiquitous enzyme produced by commensal flora and opportunistic pathogens of the lungs [21] as well as by host immune cells in normal and pathological conditions [22–26]. We already validated this enzymatic method for detecting pathogens causing pulmonary infections [27], and for improving the detection of lung biomarkers [28–30]. Here it will be shown that it can be used to prepare sputum samples for cytometric analysis as well. Following our protocol for enzymatically liquefying respiratory samples the single-cell suspensions required for flow cytometry are obtained more rapidly and efficiently than with the traditional reducing procedure. Furthermore, the proposed enzymatic method could be used to address functional analysis of sputum-resident leukocytes, since it leaves the cells viable and widely untouched (Fig. 1B). The results shown here pave the way for a better profiling of pathological cellular states in lung leukocytes, which could improve the diagnosis and drive the development of targeted therapeutics in airways diseases.

### Materials and methods

#### Respiratory samples collection

Forty-five expectorated sputum samples were collected from June 2021 to May 2022 by the Department of Microbiology from Son Espases University Hospital (Balearic Islands). The study was conducted according to the ethical guidelines of the 1975 Declaration of Helsinki. A microscopic screening was performed to determine their suitability for being included in the study. The inclusion criteria were; (i) sputum expectorated within last 24 h, (ii) a positive result in the Gram’s stain test, and (iii) the presence of ≥ 25 polymorphonuclear white blood cells and < 10 squamous epithelial cells per field (at 100 × total magnification). All samples were anonymized leftover specimens that, otherwise, would have been discarded. They were obtained in the clinical routine practice from patients with suspected pulmonary infection. Any leftovers were destroyed. For these reasons the institutional review board considered the study as minimal-risk research and waived the requirement for informed consent (Ethics and Scientific Committee approval with reference IB 4005/19-PI).

#### Liquefaction of sputum samples

120 mg of each sputum sample was weighed out in triplicate using a HR-150AZ analytical balance (e = 0.001 g) and collected in conical 15 mL polypropylene tubes. Then, sputum samples were liquefied following the gold standard method based on using dithiotreitol (DTT) as reducing agent, or our enzymatic method based on adding H_2_O_2_. Briefly, for the reducing method sputum samples were liquefied by adding 6.5 mM DTT (Invitrogen) in PBS at a 10:1 constant ratio (v/w) for 30 min at 37 ºC with 10-min intervals of vigorous vortex mixing. For the enzymatic method sputum samples were liquefied by adding 0.3 M H_2_O_2_ (Invitrogen) in PBS at a 10:1 constant ratio (v/w) for 60–120 s at room temperature (RT). Additionally, sputum samples treated with PBS at a 10:1 constant ratio (v/w) for 30 min at RT with 10-min intervals of vigorous vortex mixing were included as controls. 200 µL of liquefied sputum samples was collected in 1.8 mL cryotubes and kept at -20 °C until turbidimetry evaluation. The remaining solutions of liquefied sputum samples were subsequently washed by centrifugation at 1700 rpm for 5 min after adding 10 mL flow cytometry buffer (FCB); PBS supplemented with 2% heat inactivated fetal bovine serum (Sigma-Aldrich) and 1 mM ethylene diamine tetraacetic acid (EDTA, Sigma-Aldrich). After supernatants removal, cell pellets from liquefied sputum samples were resuspended in 1 mL FCB and then filtered through 50 µm cup-type cell strainers (Becton Dickinson) in 1.5 mL Eppendorf tubes. The resulting cell suspensions account for the cellular content of 100 mg of liquefied sputum.

#### Microscopy for cell counting and viability

Cell density from liquefied sputum suspensions was adjusted for appropriate cell counting in a Neubauer chamber (BlauBrand). 10 µL of previously adjusted cell suspensions in FCB was added to 10 µL 0.4% Trypan blue solution (Sigma-Aldrich), then two counting grids of the Neubauer chamber were filled and the viable/dead cell counts were performed in duplicate in a bright field light microscope (Leica). Cell counts were calculated as follows; [Sputum cells·mg^−1^ = A (cell count mean of two grids) × B (cell density dilution factor) × 2 (Trypan blue dilution factor) × 10 (v/w liquefaction factor)].

### Flow cytometry

#### Cell surface staining protocol

Cell suspensions from liquefied sputum samples were centrifuged at 1500 rpm for 5 min, and then a cell membrane staining protocol was conducted. Briefly, cell pellets were resuspended with 100 µL FcR blocking solution (FCB with 1 µg·mL^−1^ human IgG from Abcam) and incubated on ice for 15 min. Next, the following mixture of fluorochrome-conjugated antibodies was added immediately without washing; mouse anti-human CD63-PCy7 (IgG1k, clone H5C6 from Becton Dickinson), mouse anti-human HLA-DR-APC/Fire 810 (IgG2ak, clone L243), mouse anti-human CD45-APC (IgG1k, clone 2D1), mouse anti-human CD11b-FITC (IgG1k, clone ICRF44), mouse anti-human CD206-PE (IgG1k, clone 15–2) and mouse anti-human CD16-PCy5 (IgG1k, clone 3G8), all purchased from Biolegend. Then, 2 µL of DAPI solution (Becton Dickinson) previously diluted 1:20 in FCB was added and cell suspensions were placed on ice for 20 min. After incubation, cells were washed once by adding 1 mL FCB and centrifuged for 5 min at 1500 rpm. The mouse IgG1k-PCy7 isotype control (clone P3.6.2.8.1 from Becton Dickinson) was included in order to evaluate the non-specific binding of fluorescent IgG antibodies. Finally, stained cells were resuspended with 300 µL FCB and acquired on a BD FACSVerse flow cytometer (Becton Dickinson) equipped with 488, 640 and 405 nm lasers. Fluorescence compensation was performed by acquiring single-stained leukocytes for each fluorochrome and using the automated compensation tool in FACSuit software v1.0.5.3841 (Becton Dickinson). All flow cytometry data was subsequently analyzed with FlowJo software v10.6 (Becton Dickinson). Cellular autofluorescence measurements (see Table S1) were performed in DAPI‾ viable cells following the abovementioned protocol without conducting the blockade of FcR nor the addition of fluorochrome-conjugated antibodies.

#### Phenotyping and activation status of sputum leukocytes after liquefaction

Cells were gated in a FSC-H/FSC-A dot-plot to eliminate doublets and viable cells were selected by excluding DAPI dye (Fig. 4A and B). Then, total CD45^High^ leukocytes were gated in a SSC-A/CD45 dot-plot (Fig. 4C). Granulocytes and lymphocytes were selected in a subsequent gate by excluding CD206 fluorescence (macrophages and dendritic cells marker) (Fig. 4D). Next, in a SSC-A/CD11b dot-plot SSC^Low^CD11b^Low/‾^ total lymphocytes and SSC^High/+^CD11b^+^ total granulocytes were identified (Fig. 4E). Then, granulocytes were plotted in a CD16/CD11b dot-plot and two gates were defined, one for the selection of CD16^High^CD11b^+^ mature neutrophils and another one including CD16^Low^CD11b^+^ neutrophils and CD16‾CD11b^+^ eosinophils (Fig. 4F). Finally, eosinophils were definitely identified as SSC^High^ cells in a subsequent SSC-A/FSC-A dot-plot (Fig. 4G). Frequency of total lymphocytes was referred to CD45^High^CD206^‾^leukocytes and frequencies of CD16^High^CD11b^+^ mature neutrophils and CD16‾CD11b^+^SSC^High^ eosinophils were referred to total granulocytes. The median fluorescence intensity (MFI) of HLA-DR (activation marker), CD63 (degranulation marker) and CD11b (leukocyte adhesion marker) was evaluated in order to analyze the impact of the liquefaction methods on the activation status of sputum leukocytes subpopulations (histograms in Fig. 4E-G). A minimum of 100 events in each gate were analyzed in order to ensure the accuracy of the data.

### Turbidimetry

One hundred μL of all liquefied sputum samples was placed in 96-well ELISA plates (Thermo Fisher Scientific). Then, the absorbance at 800 nm was measured using a PowerWave HT plate reader (Biotek). The absorbance was evaluated in triplicate with 30 s of gentle manual shaking between determinations, in order to evaluate the impact of the degree of liquefaction on the variability of the measurement. The transmittance was calculated as the inverse of the absorbance at 800 nm.

### Statistical analysis

Statistical analysis was performed using the GraphPad Prism software (San Diego, USA). Kruskall-Wallis and Mann–Whitney tests were used, respectively, to compare differences between three liquefying procedures (the proposed enzymatic method, the traditional reducing procedure and PBS control) and two groups of derived data (ratios treatment/PBS). A p value < 0.05 was considered statistically significant.

## Results

### Degree of sputum liquefaction and impact on cell recovery and non-specific binding of fluorescent antibodies

The present study aims to demonstrate the suitability of a novel enzymatic method to prepare respiratory samples for single-cell analysis by flow cytometry. In previous works we have demonstrated that catalase generates oxygen bubbles that liquefies low respiratory tract specimens (e.g., bronchial aspirates and sputum) in 60 s after the addition of 0.3 M H_2_O_2_ [27–29]. Moreover, we previously demonstrated that H_2_O_2_ does not reduce the disulfide bonds cross-linking mucins in the respiratory samples, in contrast to the traditional reducing DTT reagent [29]. We also proved that catalase is essential to liquefy the samples, since in the presence of catalase inhibitors H_2_O_2_ cannot dissolve the mucin matrix [27]. Thus, the main liquefying factor in our enzymatic method is the generation of bubbles that mechanically disrupts the matrix (Fig. 1A and Figure S1). Since the proposed enzymatic method is faster and requires less instrumentation (i.e., cell handling is more appropriate) than the traditional DTT procedure, we envisioned that it could overcome the issues related to flow cytometry analysis of sputum-resident leukocytes (Fig. 1B).

**Fig. 2:**
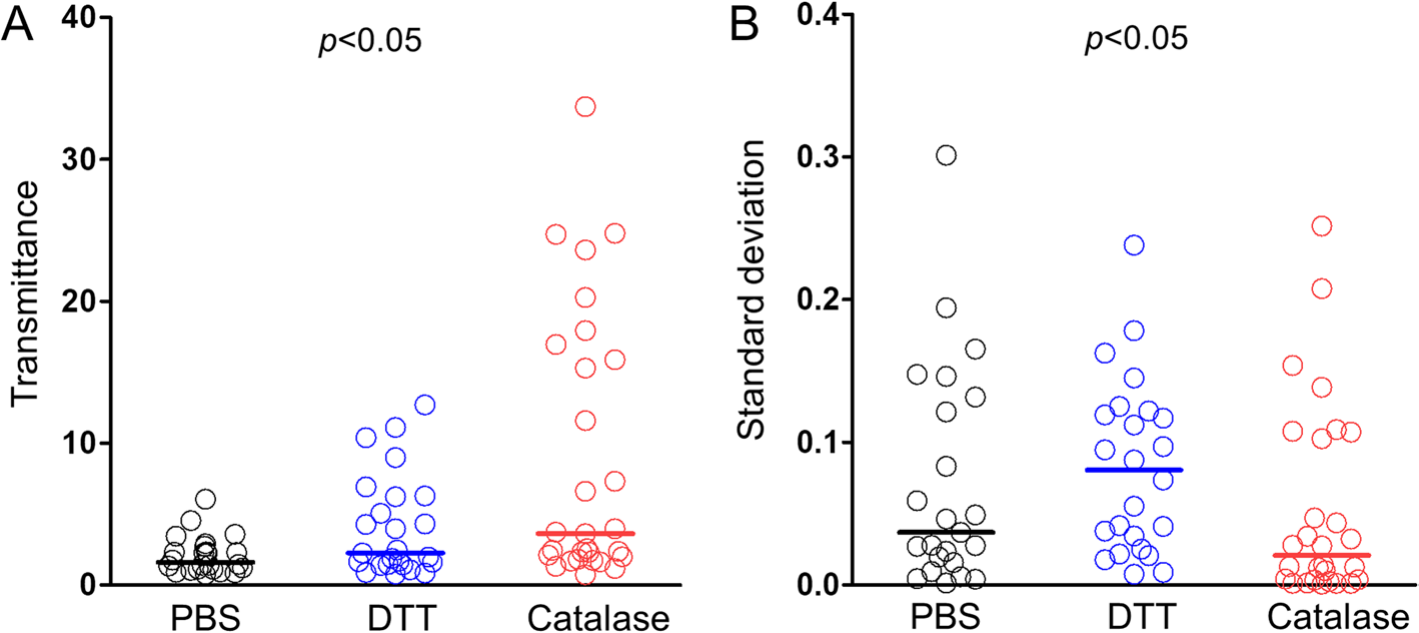
Sputum liquefaction degree after the proposed enzymatic method and the traditional reducing procedure. Transmittance averages (**A**) and standard deviations (**B**) obtained from 3 absorbance measures at 800 nm of sputum samples (*n* = 29) processed with PBS (black), the traditional DTT procedure (blue) and the proposed enzymatic method (red). Horizontal bars represent the median and *p*-values were yielded by a Kruskall-Wallis test

First, we evaluated the degree of liquefaction after using the enzymatic method or DTT by measuring the transmittance in different samplings of the same sputum. In Fig. 2A, sputum samples have been liquefied to a larger extent by the enzymatic procedure, because they yield higher transmittance values, that is, they are more transparent. The distribution and size heterogeneity of particles within the samples influence the light dispersion and, accordingly, the variability (standard deviation) of 3 repeated determinations of transmittance is higher in sputum samples that are poorly liquefied by DTT or PBS (Fig. 2B). These results demonstrate that the enzymatic method homogenizes sputum samples better than the traditional reducing method.

**Fig. 3:**
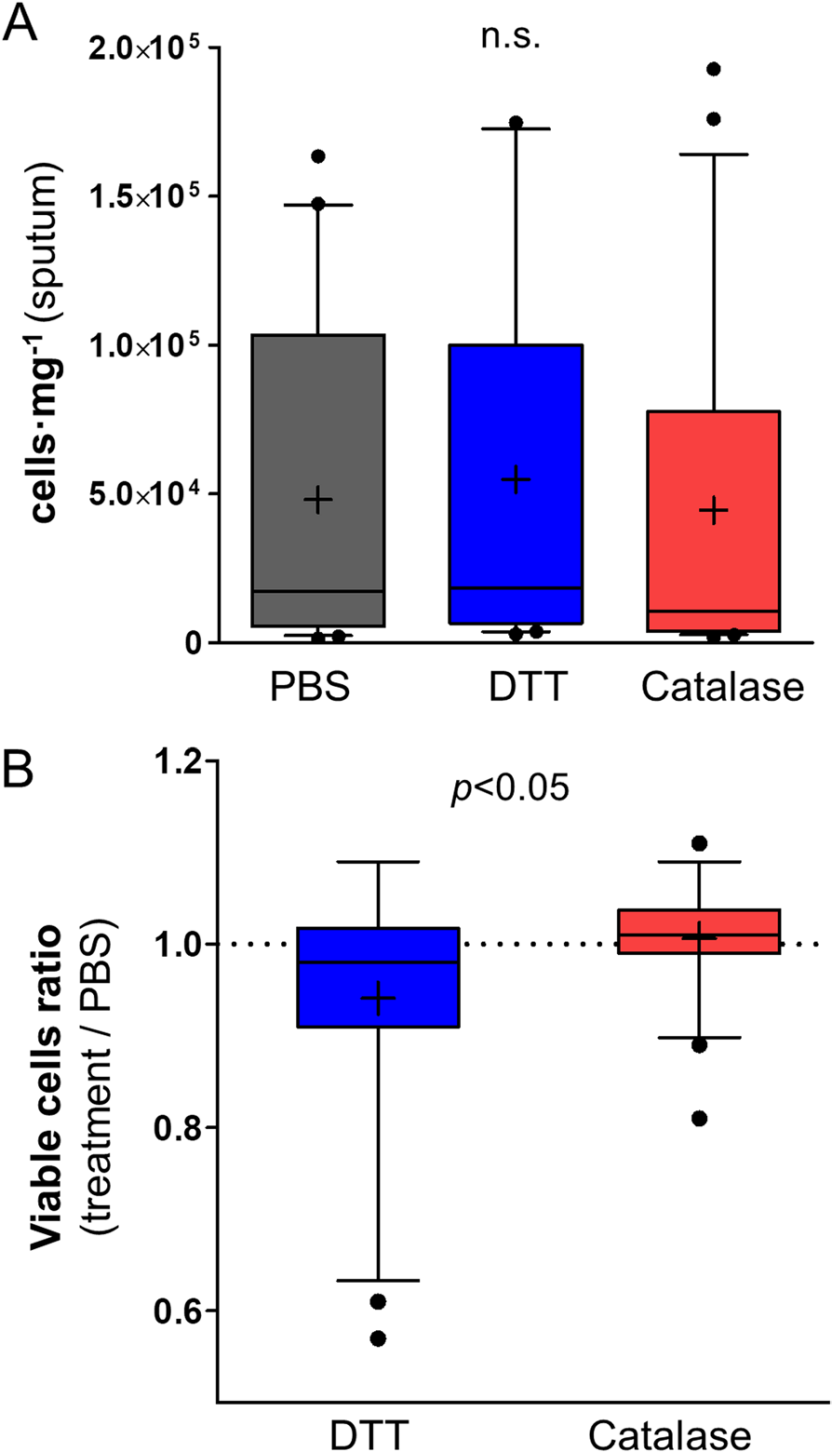
Absolute cell numbers and cell viability in sputum samples liquefied by the enzymatic and the reducing procedure. Cell counts (**A**) and treatment-to-PBS ratios of viable cell counts (**B**) in sputum samples (*n* = 21) processed with PBS (black), the traditional DTT procedure (blue) and the proposed enzymatic method (red). Boxes represent medians (horizontal bars) and means (crosses) with 25th-75th percentiles, and wishkers represent 10th-90th percentiles. *p*-values were yielded by a Kruskall-Wallis test in (**A**) and a Mann–Whitney test in (**B**). n.s.; non-significant

Next, we evaluated if the higher degree of liquefaction yielded by the enzymatic method has a positive impact in the cell recovery from liquefied sputum samples. For this purpose, we conducted absolute cell counts and evaluated cell viability in liquefied sputum samples using a Neubauer chamber and a trypan blue exclusion test. In Fig. 3A, total sputum cell counts are similar regardless of the method used for liquefying samples. However, the viability of sputum cells is lower in samples liquefied with DTT than in those liquefied with the enzymatic method (Fig. 3B). These results prove that while cell recovery in enzymatically liquefied sputum samples is comparable to that after liquefaction with the traditional DTT procedure, the proposed method is better at preserving cell viability, which may facilitate their subsequent cytometric analysis. Furthermore, it reduces the non-specific binding of fluorescent antibodies to cell membranes as shown in Figure S2. In these experiments, the MFI of the IgG1k-PCy7 isotype control (5 of 6 fluorescent antibodies used are IgG1k) is higher in those samples liquefied to a lesser extent by DTT (right in Figure S2), which indicates that the enzymatic methods reduces the background signal thanks to its higher liquefaction efficiency.

### Impact of liquefaction method on the cytometric analysis of lung leukocytes

**Fig. 4:**
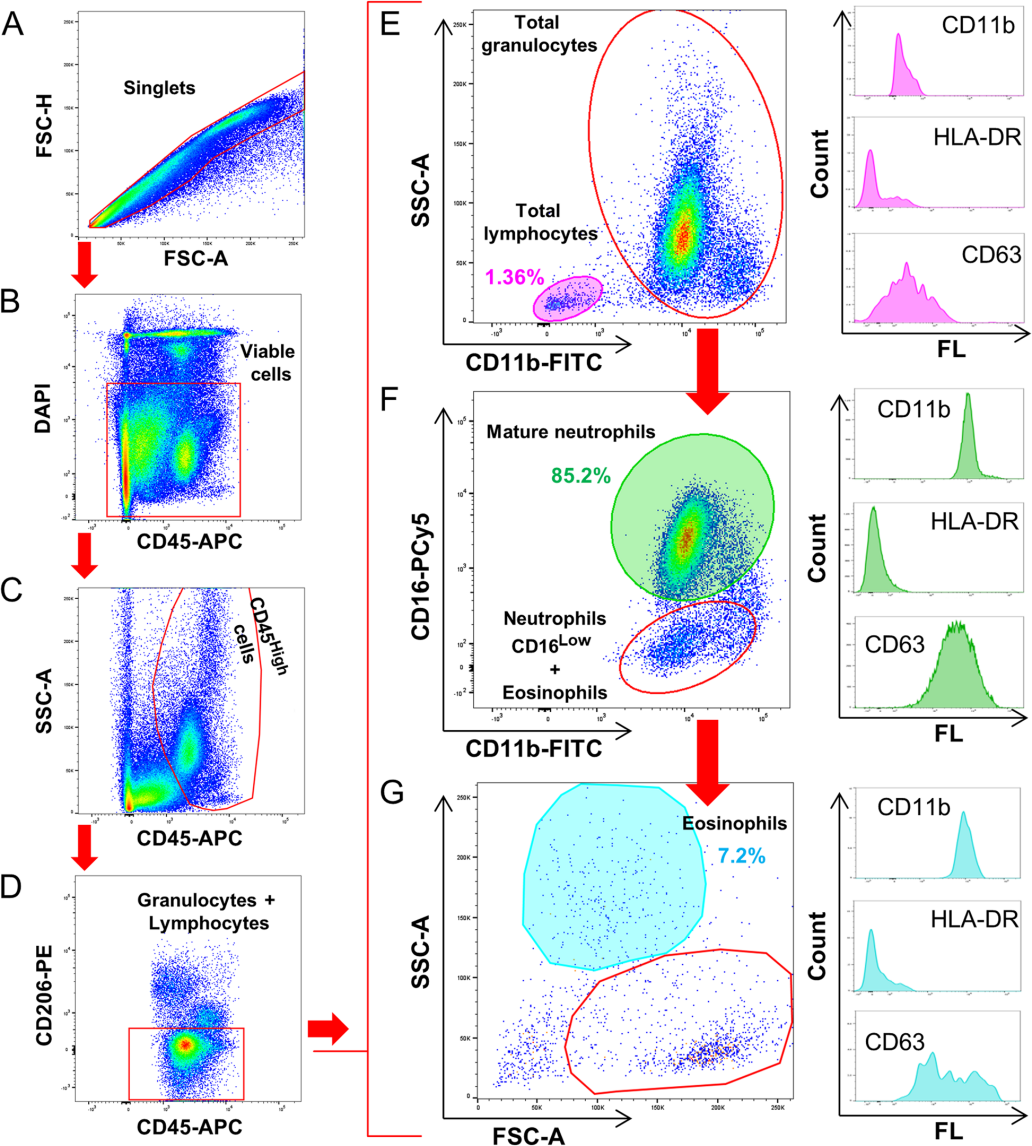
Cytometric gating strategy for the evaluation of frequencies and activation status of sputum leukocytes. Singlets (**A**), viable cells (**B**), total leukocytes (**C**) and total granulocytes/lymphocytes (**D**) are sequentially selected and then total lymphocytes and granulocytes are plotted in a SSC-A/CD11b-FITC dot-plot (**E**). Next, total granulocytes (red gate in E) are plotted in a CD16-PCy5/CD11b-FITC dot-plot and gates for selecting CD16^High^ mature neutrophils and CD16^Low^ neutrophils + eosinophils are defined (**F**). Finally, CD16^Low^ neutrophils + eosinophils (red gate in F) are plotted in a SSC-A/FSC-A dot-plot and SSC^High^ eosinophils are selected (**G**). Cell frequencies and the MFI of membrane markers (percentages and histograms, respectively, in panels **E** to **G**) are evaluated in total lymphocytes (pink) mature neutrophils (green) and eosinophils (blue) gates

**Fig. 5:**
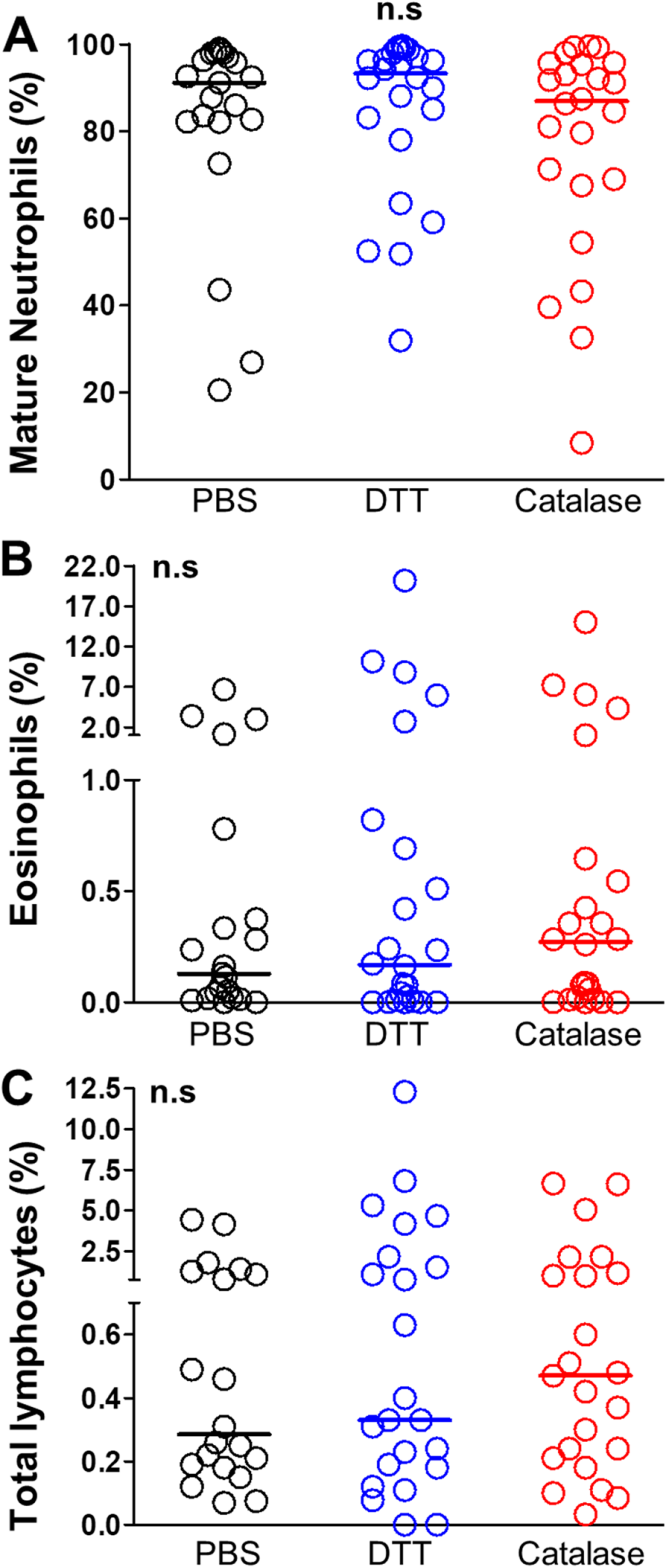
Frequencies of sputum leukocytes after liquefaction with the proposed enzymatic method and the traditional reducing procedure. Frequencies (%) of sputum-resident mature neutrophils (**A**), eosinophils (**B**) and total lymphocytes (**C**) in sputum samples (*n* = 24) processed with PBS (black), the traditional DTT procedure (blue) and the proposed enzymatic method (red). Horizontal bars represent the median and *p*-values were yielded by a Kruskall-Wallis test. n.s.; non-significant

Next, we determined whether the liquefying method used to prepare sputum samples could have any impact on the flow cytometry analysis of lung leukocytes. To this end, we conducted a comparative study of the frequencies of sputum-resident neutrophils, eosinophils and lymphocytes detected in DTT- and enzyme-liquefied samples. The gating strategy and phenotyping of sputum leukocytes are depicted in Fig. 4A-G. As expected, mature neutrophils (identified as CD45^High^CD206‾CD11b^+^CD16^High^ cells) were detected in all liquefied sputum samples and accounted for the most frequent leukocyte subset (Fig. 4F), without statistical differences between the enzymatic and the traditional reducing method or PBS (Fig. 5A). Sputum-resident lymphocytes (identified as CD45^High^CD206‾CD11b^Low/‾^SSC^Low^ cells) and eosinophils (identified as CD45^High^CD206‾CD11b^+^CD16‾SSC^High^ cells) were the least abundant cell subsets in liquefied samples (Fig. 4E and G, respectively) and, again, their frequencies were similar regardless of the liquefying method used (Fig. 5B-C). Nevertheless, Figure S3 shows a more accurate cytometric analysis in sputum samples liquefied by the enzymatic method, due to a more reliable delineation of the gates for selecting total CD45^High^ leukocytes (Figure S3A) and discriminating lymphocytes from total granulocytes (Figure S3B). In addition, the enzymatic liquefaction circumvents the visualization of artefacts that may interfere with optimal flow cytometry data (samples S2, S4 and S5 in Figure S3A). These results demonstrate that the proposed enzymatic method for liquefying sputa properly prepare these respiratory samples for subsequent single-cell cytometric analysis.

### Enzymatic liquefaction of sputum samples leaves lung leukocytes untouched

**Fig. 6:**
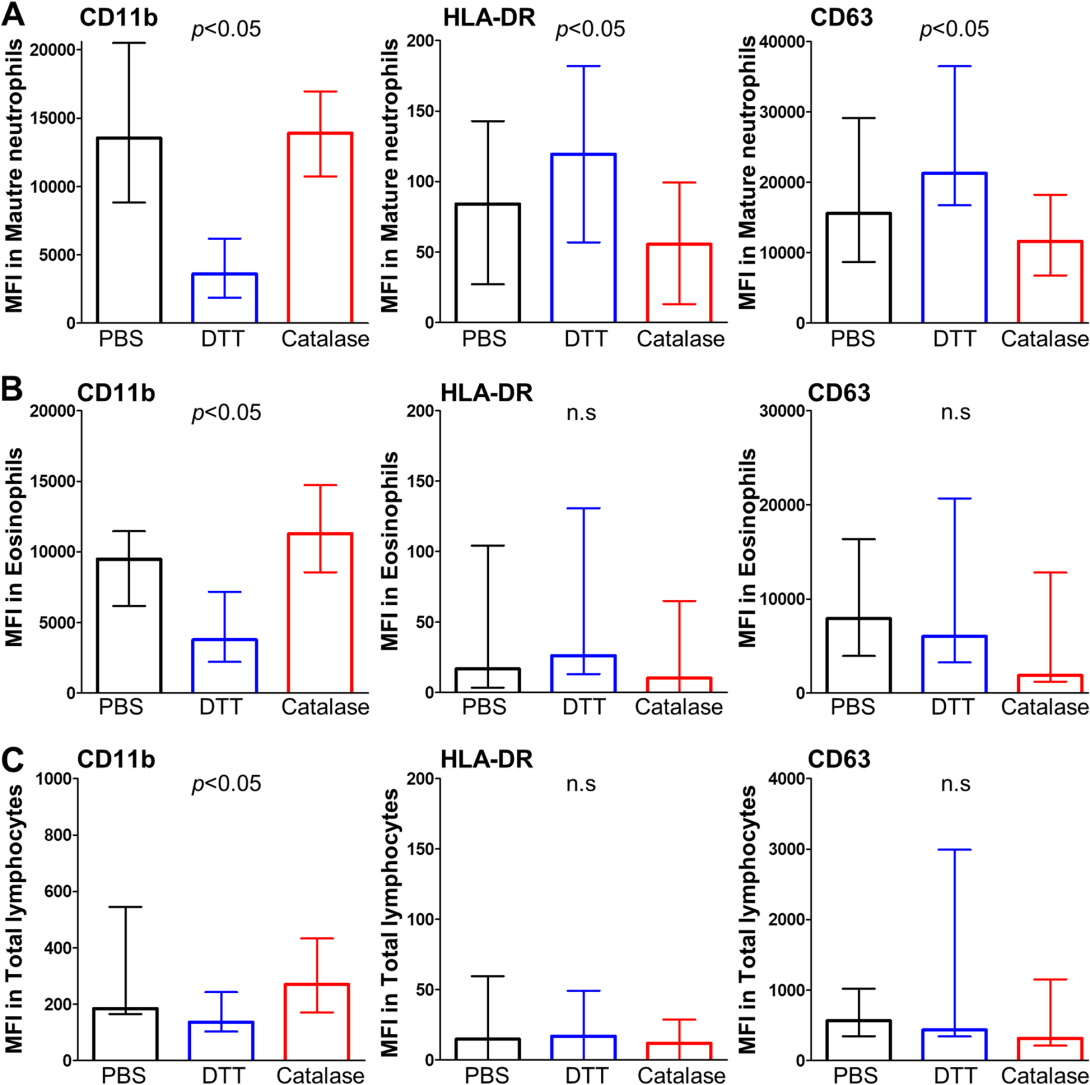
Activation status of leukocytes in enzyme- and DTT-liquefied sputum samples. Expression levels (MFI) of CD11b, HLA-DR and CD63 in sputum-resident mature neutrophils (**A**, *n* = 20), eosinophils (**B**, *n* = 10) and total lymphocytes (**C**, *n* = 15) after processing sputum samples with PBS (black), the traditional DTT procedure (blue) and the proposed enzymatic method (red). Horizontal bars represent the median and error bars the 25th-75th percentiles. *p*-values were yielded by a Kruskall-Wallis test. n.s.; non-significant

We already demonstrated that the cell viability in sputum samples is mostly unaffected by the enzymatic liquefaction (Fig. 3B). This evidence supports the idea that sputum-resident leukocytes in enzyme-liquefied samples are untouched. However, to properly evaluate the local immune response in the airways (e.g., against pulmonary infections) the pre-processing of respiratory samples should not influence the functionality of lung leukocytes either. Accordingly, we purposed to compare the cell activation induced by the liquefying methods used in this study to prepare sputum samples for flow cytometry. First, we discarded differences in autofluorescence levels yielded by viable cells (DAPI‾) in DTT- and enzyme-liquefied sputum samples (Table S1). Furthermore, hydrogen peroxide does not hamper the detection of CD11b, HLA-DR and CD63 even in the absence of antioxidant activity of sputum matrix-derived catalase (Figure S4). Then, we compared MFI levels of CD11b, HLA-DR and CD63 membrane markers in total lymphocytes, mature neutrophils and eosinophils (Figs. 4E-G) from sputum samplings processed in parallel with DTT, the enzymatic method or PBS. In Fig. 6, the expression of leukocyte adhesion marker CD11b is remarkably decreased in all subsets of leukocytes analyzed after liquefying sputum specimens with DTT, whereas no differences were found between the enzymatic method and the PBS treatment (left panels in Fig. 6A-C). Moreover, the traditional DTT procedure induces higher levels of HLA-DR (activation marker) and CD63 (degranulation marker) than the enzymatic method in sputum-resident mature neutrophils (middle and right panel in Fig. 6A, respectively). HLA-DR and CD63 expression levels in eosinophils and lymphocytes obtained from enzyme-liquefied sputum samples tend to be lower than those from DTT- and PBS-treated samples (middle and right panels in Fig. 6B-C, respectively), although differences do not reach statistical significance. All together, these results demonstrate that sputum-resident leukocytes remain widely untouched after enzymatic liquefaction of samples, given that changes in their activation status are not recognized.

## Discussion

In this manuscript, we conducted a comparative flow cytometry study of sputum-resident leukocytes after liquefaction with an enzymatic method, the traditional reducing procedure with DTT, and PBS buffer. Protocols for sputum liquefaction with DTT and PBS involve 30 min of incubation with cycles of vigorous vortexing (in a temperature-controlled bath in the case of DTT). Our proposed method liquefies the sample in 60 s without needing any additional instrumentation to disperse it. In this method, liquefaction is conducted by O_2_bubbles generated via enzymatic conversion of hydrogen peroxide by endogenous catalase within the samples [27–29].

The enzymatic method has some notable strengths compared to the traditional DTT, which positively impact on the subsequent cytometric analysis of sputum-resident leukocytes. First, the rapid turnaround time for liquefying sputum samples [27–29] and the ubiquitous presence of antioxidant catalase enzymes within them [21–26], circumvent the potential risk of adding hydrogen peroxide for the cell recovery and viability (Fig. 3). In addition, given that the enzymatic approach homogenizes sputum samples better than the reducing method with DTT (Fig. 2), it reduces cell clumping within single-cell suspensions, which is critical for successful cytometric analysis. Indeed, PBS- and DTT-liquefied sputum samples usually required additional filtration steps with cell strainers for an optimal sample flow through the fluidic system of the cytometer (data not shown). The better dissolution of sputum matrices after the enzymatic method also decreases the fluorescent signal from the non-specific binding of fluorescent antibodies to cell surface (Figure S2), and autofluorescence values are comparable to those obtained after DTT liquefaction (Table S1). Moreover, the accuracy during the delineation of the gates for selecting cell subsets it is also better after the enzymatic liquefaction procedure (Figure S3). Altogether, these findings demonstrate that the liquefaction of sputum samples with our enzymatic method improves the analysis or sputum samples by flow cytometry compared to the traditional reducing DTT.

When comparing frequencies of mature neutrophils, eosinophils and lymphocytes no differences were found between enzyme-, DTT- and PBS-liquefied sputum samplings of the same specimen (Fig. 5). However, it is worth mentioning that our enzymatic method is capable of liquefying highly viscous respiratory samples (e.g., bronchial aspirates) displaying liquefaction resistance if using DTT [28], that could be critical for the flow cytometry analysis of this type of samples. Furthermore, the enzymatic method does not influence the surface expression (MFI) of any of the membrane markers used in this study for assessing the activation status of sputum-resident leukocytes. Conversely, after the liquefaction with DTT the MFI of HLA-DR and CD63 is markedly increased in mature neutrophils (that is they are activated) and the MFI of CD11b is decreased in all cell subsets analyzed (Fig. 6). It is known that CD11b/CD18 complex is rapidly endocytosed from the neutrophils surface upon activation [31]. However, it cannot be ruled out that conformational changes induced by DTT-mediated reduction of disulfide bonds within CD11b protein structure could be hampering the recognition of the epitope by the specific fluorochrome-conjugated antibody used for its detection [28, 32]. Moreover, reducing and reforming of disulfide bonds in ectodomains of membrane integrins (i.e., CD11b/CD18 complex) is a recognized mechanism for activating/deactivating many cellular pathways (e.g., cell metabolism, gene expression, inflammation, etc.) [33]. The enzymatic method does not reduce disulfide bonds [29] and therefore it is more advantageous than DTT for sustaining the recognition sites of antibodies used in flow cytometry applications and retaining in vivo functional characteristics of cells. Thus, the comparative flow cytometry study performed here aligns with previous works reporting the detrimental effect of DTT on cell functionality and surface marker detection [18–20] and, additionally, demonstrates that these issues can be circumvented with the proposed enzymatic method.

## Conclusion

In conclusion, in this manuscript we validate a simple and rapid enzymatic method to liquefy sputum samples and prepare them for flow cytometry analysis. This method disperses the sputum matrix better than DTT and handles cells in a gentler way. These hallmarks provide a more accurate cytometric analysis and leave sputum-resident cells widely untouched, which benefits the study of the cellular basis of pathological processes in the lungs.

## Supplementary Information

**Additional file 1. **The following associated content is available online in the supporting information datasheet: Nanoparticle tracking analysis of artificial sputum treated with DTT or H2O2 in the absence of catalase (Figure S1); Non-specific binding of fluorescent IgG antibodies to sputum leukocytes depending on the sample liquefaction degree (Figure S2); Optimized cytometric analysis of sputum leukocytes after enzymatic liquefaction of samples (Figure S3); Impact of hydrogen peroxide on CD11b, HLA-DR and CD63 detection in blood leukocytes (Figure S4). Autofluorescence in DAPI-negative leukocytes from liquefied sputum samples (Table S1).

## Data Availability

The flow cytometry data that support the findings of this study are openly available in “Comparative study of sputum-resident leukocytes” at http://flowrepository.org (repository ID: FR-FCM-Z5MK).
