# Supplementary material for: Improved cytometric analysis of untouched lung leukocytes by enzymatic liquefaction of sputum samples
Source: Biol Proced Online. 2022 Nov 17;24:17. doi: 10.1186/s12575-022-00181-z (PMC9673301; doi:10.1186/s12575-022-00181-z)
Supplement: Supplementary file 1 — Additional file 1. The following associated content is available online in the supporting information datasheet: Nanoparticle tracking analysis of artificial sputum treated with DTT or H2O2 in the absence of catalase (Figure S1); Non-specific binding of fluorescent IgG antibodies to sputum leukocytes depending on the sample liquefaction degree (Figure S2); Optimized cytometric analysis of sputum leukocytes after enzymatic liquefaction of samples (Figure S3); Impact of hydrogen peroxide on CD11b, HLA-DR and CD63 detection in blood leukocytes (Figure S4). Autofluorescence in DAPI-negative leukocytes from liquefied sputum samples (Table S1). [file 12575_2022_181_MOESM1_ESM.docx]

**Supporting information for:**

**Improved cytometric analysis of untouched lung leukocytes by enzymatic liquefaction of sputum samples**

Giulia Santopolo,^1,2^ Antonio Clemente,^1,*^ Estrella Rojo-Molinero,^3,4^ Sara Fernández^3^, María Concepción Álvarez^3^, Antonio Oliver^3,4^ and Roberto de la Rica^1,4^

^1^Multidisciplinary Sepsis Group, Hospital Universitario Son Espases, Health Research Institute of Balearic Islands (IdISBa), Palma de Mallorca, Spain

^2^Department of Chemistry, University of the Balearic Islands, Palma de Mallorca, Spain

^3^Microbiology Department, Hospital Universitario Son Espases, Health Research Institute of Balearic Islands (IdISBa), Palma de Mallorca, Spain

^4^CIBER de Enfermedades Infecciosas (CIBERINFEC), Madrid, Spain

**Running head:** Cytometric analysis of enzyme-liquefied sputa

***Correspondence to:** [antonio.clemente@ssib.es](mailto:antonio.clemente@ssib.es)

**Contents:**

**Figure S1.** Nanoparticle tracking analysis of artificial sputum treated with DTT or H_2_O_2_ in the absence of catalase.

**Figure S2.** Non-specific binding of fluorescent IgG antibodies to sputum leukocytes depending on the sample liquefaction degree.

**Figure S3.** Optimized cytometric analysis of sputum leukocytes after enzymatic liquefaction of samples.

**Figure S4.** Impact of hydrogen peroxide on CD11b, HLA-DR and CD63 detection in blood leukocytes.

**Table S1.** Autofluorescence in DAPI-negative leukocytes from liquefied sputum samples.

**S1. Nanoparticle tracking analysis**

We used nanoparticle tracking analysis (NTA), which estimates the dimensions of nano-sized objects based on tracking their brownian motion trajectories, for evaluating the impact of reducing DTT reagent and hydrogen peroxide on the polymeric mucin assembly in the absence of catalase enzymes. Briefly, 5 mg mucin (M2378 reference from Sigma-Aldrich) was weighed in a 1.5 mL Eppendorf tube, 1 mL PBS was added and the resulting solution was kept overnight at 4 ºC to favor mucin polymerization. Next, 100 μL mucin solution was added to 1 mL PBS or DTT (Sigma-Aldrich) solution (6.5 mM in PBS) and then incubated for 30 min at room temperature (RT). For hydrogen peroxide treatment 100 μL mucin solution was added to 900 μL PBS, incubated for 29 min at RT and then 100 μL 3 M hydrogen peroxide (Sigma-Aldrich) was added for 1 min at RT. Finally, all samples were diluted 1:5 in Milli-Q water before the injection in the low volume flow cell of a Nanosight NS300 (Malvern Panalytical). Measures were taken in triplicate with a recording time of 120 s.

In Figure S1, particle size distribution is similar between mucin samples treated with PBS (black) and hydrogen peroxide (red). In contrast, the particle size distribution is lower in mucin samples treated with DTT (blue). These results suggest that DTT reduces disulfide bonds cross-linking mucins and disrupts their polymeric assembly. However, the condensation of mucin polymers is not affected by the hydrogen peroxide treatment *per se* (without catalase). This supports the idea that oxygen bubbles produced by catalase enzymes mechanically disrupts cross-linked mucins in sputum samples treated with hydrogen peroxide. However, we cannot rule out that hydrogen peroxide entering the intermolecular gap created by the oxygen bubbles oxidizes the mucin molecules and induces their oxidative degradation to some extent.


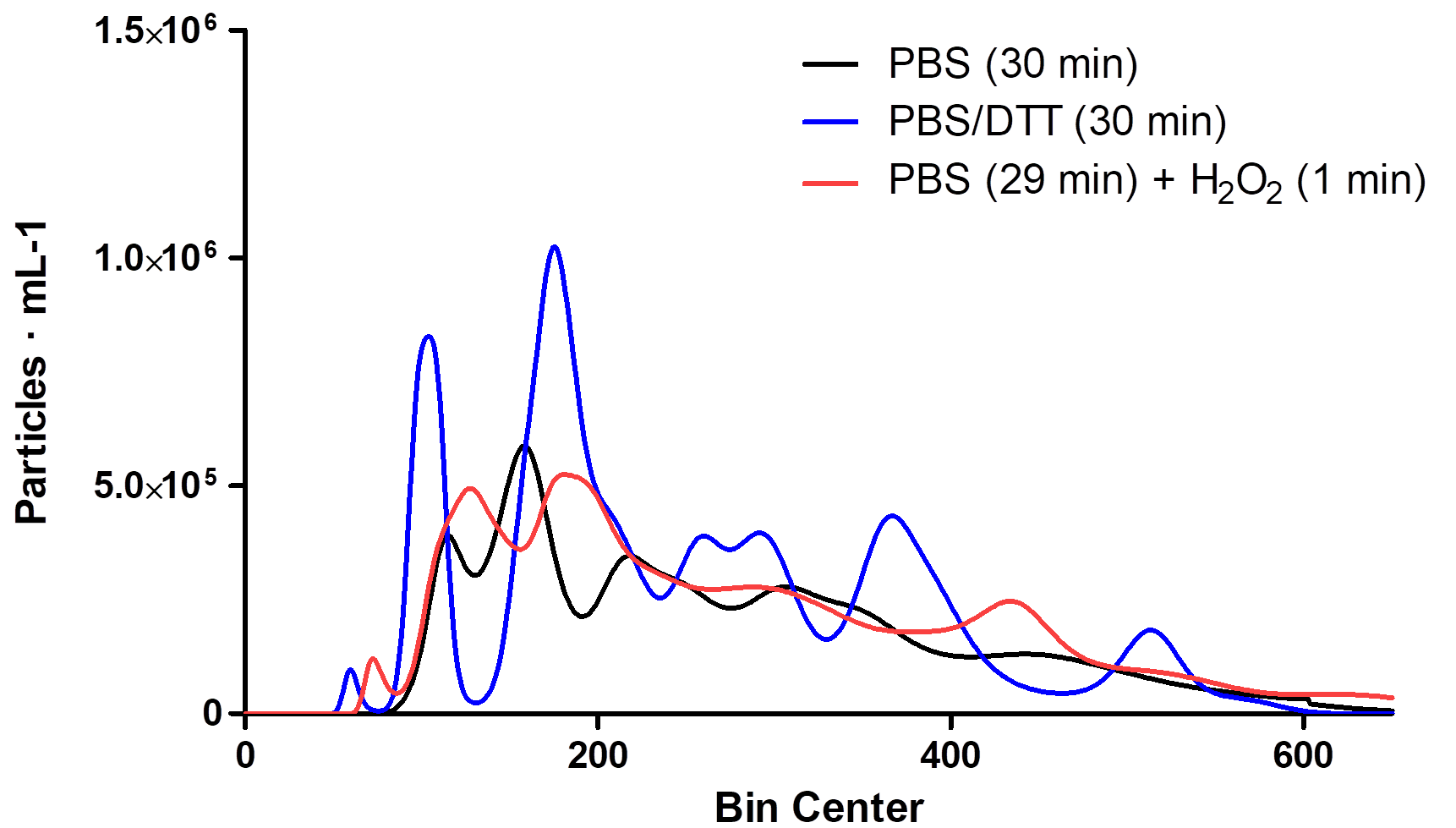


**Figure S1. Nanoparticle tracking analysis of artificial sputum treated with DTT or H_2_O_2_ in the absence of catalase.** Size distribution of particles in mucin samples treated with PBS (black), hydrogen peroxide (red) or DTT (blue).

**S2. Evaluation of non-specific binding of fluorescent IgG antibodies to sputum leukocytes**

We evaluated the impact of the matrix dissolution efficiency on the non-specific binding of fluorescent antibodies to leukocytes within liquefied sputum samples. To this end, different samplings of the same sputum were processed in parallel with PBS, the traditional DTT procedure and the proposed enzymatic method (see 2.2. section in the main text). Next, we measured the transmittance of the liquefied sputum samples (see 2.5. section in the main text). Then, sputum samples were classified according to the obtained liquefaction yields as explained below. First, DTT-to-PBS and catalase-to-PBS ratios of transmittance values were obtained. Thus, we considered that the liquefaction yield of enzymatic method is significantly higher when:

*[catalase-to-PBS ratio ̶ DTT-to-PBS ratio] ≥ 50 % of catalase-to-PBS ratio value*

Finally, we used an isotype IgG control conjugated with PCy7 for staining the processed sputum samples and evaluated the MFI in viable cells (see 2.4. section in the main text). MFI levels yielded by the isotype control are similar in sputum samples equally liquefied by DTT and the enzymatic method (left in Figure S2). In contrast, MFI levels are higher in sputum samples that have been liquefied to a lesser extent by DTT (right in Figure S2). These results demonstrate that the poor matrix dissolution in DTT-liquefied samples prompts the non-specific binding of fluorescent antibodies.


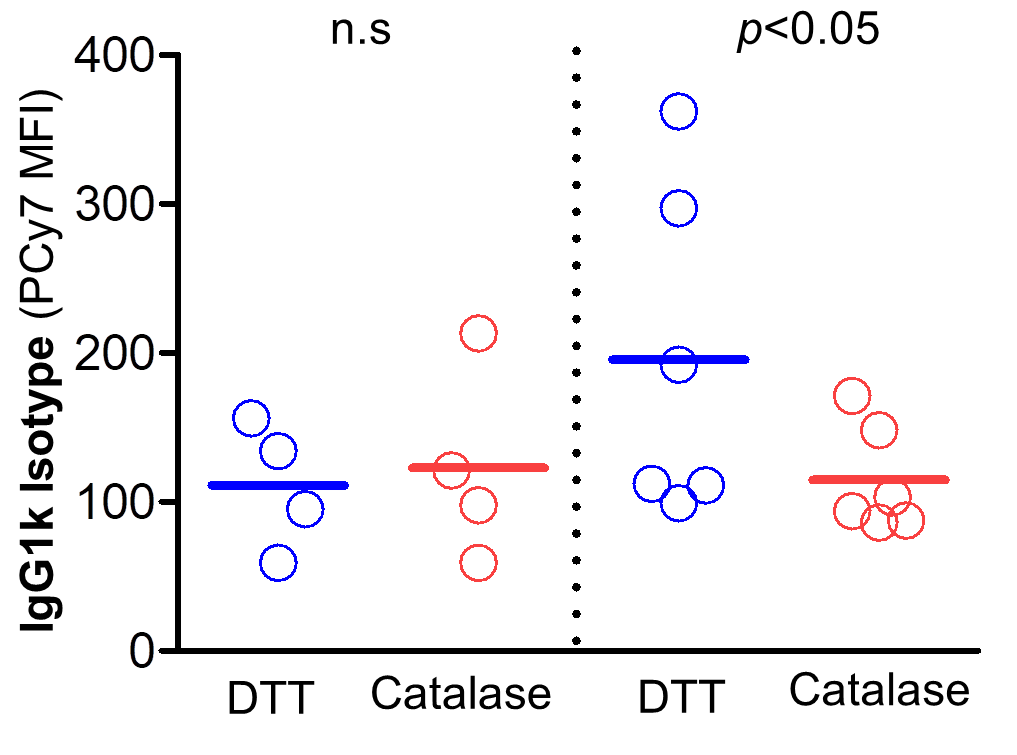


**Figure S2. Non-specific binding of fluorescent IgG antibodies to sputum leukocytes depending on the sample liquefaction degree.** MFI levels yielded by isotype control after processing sputum with DTT (blue) and the enzymatic method (red) in samples equally liquefied by both methods (left) and samples more efficiently liquefied by the enzymatic method (right). Horizontal bars represent the median and p-values were yielded by a Mann-Whitney test. n.s.; non-significant.

**
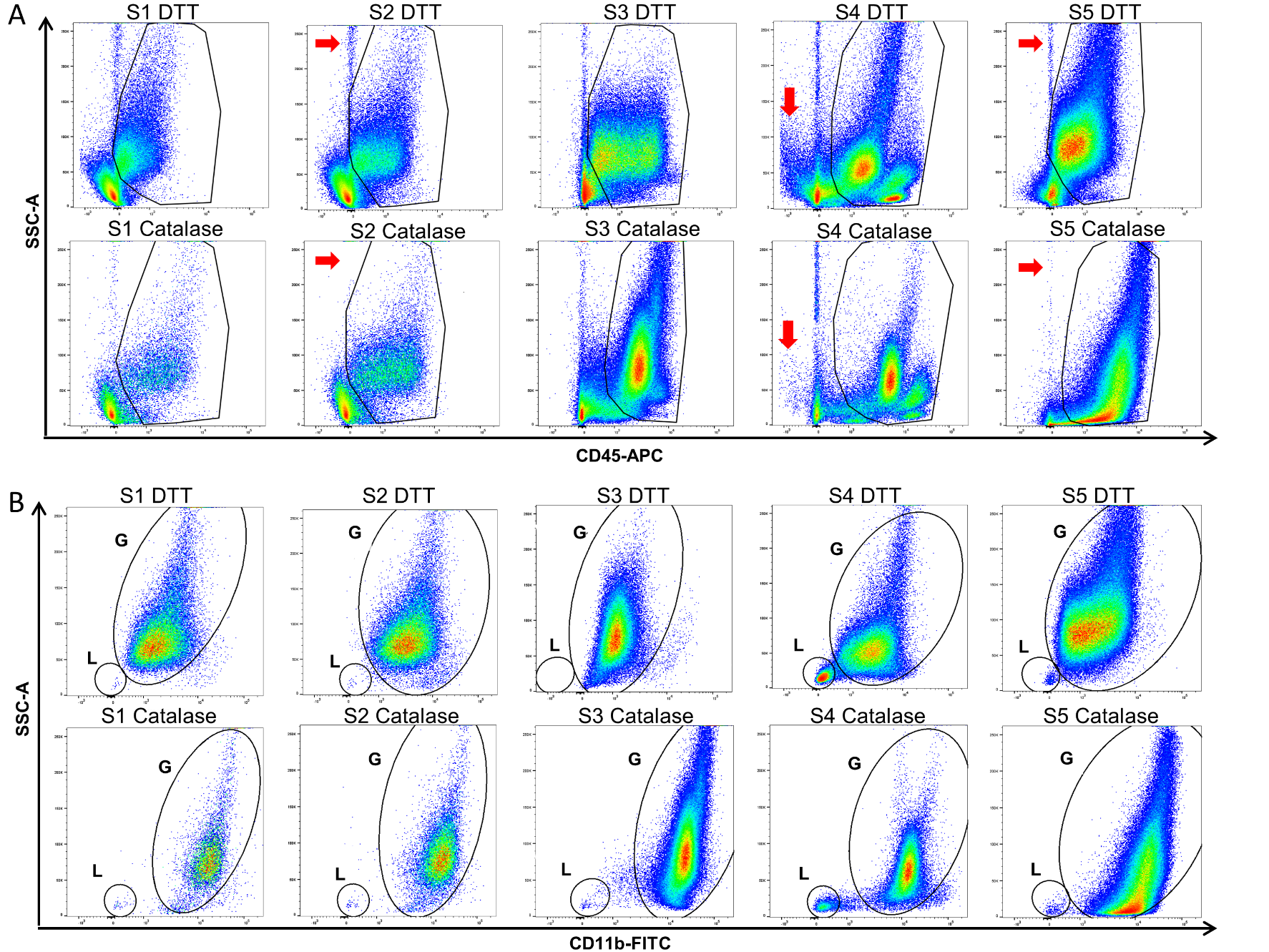
**

**Figure S3. Optimized cytometric analysis of sputum leukocytes after enzymatic liquefaction of samples.** Delineation of gates for selecting CD45High total leukocytes (A), CD11b^Low/^‾ SSC^Low^ total lymphocytes and CD11b^+^SSC^High/+^ total granulocytes (L and G gates in panel B) is more accurate in sputum samples processed with the enzymatic method (lower rows) than with DTT (upper rows). S1-S5 labels represent 5 representative liquefied sputum samples and red arrows point zones to compare cytometry artefacts depending on the liquefying method used.

**S4. Surface staining protocol for peripheral blood leukocytes**

In order to definitively discarding the detrimental effect of hydrogen peroxide on the expression of membrane markers we conducted a surface staining protocol by using leukocytes purified from peripheral blood. Briefly, 5 mL of fresh blood samples from 3 healthy volunteers were collected by venipuncture in tubes with EDTA as anticoagulant and total nucleated cells were purified by using HetaSept solution following manufacturer’s instructions (StemCell Technologies). Contaminant erythrocytes in the obtained cells pellets were removed by incubation with 2 mL of red blood cells lysis buffer (0.15 M ammonium chloride, 10 mM sodium bicarbonate, 1 mM EDTA, all from Sigma-Aldrich, pH 7.2) during 3 min in ice. After washing, cells were resuspended with 200 μL of 10 mg/mL mucin in PBS (M2378 reference from Sigma-Aldrich) and incubated during 1 hour at 4 ºC. Next, cells suspensions were split into two 15 mL conical tubes and 1 mL PBS or 0.3 M H_2_O_2_ (in PBS) was added and incubated during 2 min at RT. After incubation, cells were immediately washed with 14 mL flow cytometry buffer (FCB), centrifuged at 1700 rpm during 5 min, resuspended with 100 μL FCB and stained following the cell surface staining protocol explained in the main text. Finally, stained cells were resuspended with 300 μL FCB and acquired on a BD FACSVerse flow cytometer (Becton Dickinson). Median fluorescence intensity (MFI) of CD11b, HLA-DR and CD63 membrane markers was analyzed in blood leukocytes subpopulations (neutrophils, eosinophils and lymphocytes) following the gating strategy depicted in Figure 3 of the main text. Finally, ratios of MFI after hydrogen peroxide (MFI_H2O2_) to MFI after PBS (MFI_PBS_) were calculated.

If oxidation impacts on the detection of surface membrane markers, MFI values detected in cells treated with only PBS are expected to be higher than those in cells treated with hydrogen peroxide and, accordingly, MFI_H2O2_ to MFI_PBS_ ratios will be significantly lower than 1. Figure S4 shows that MFI_H2O2_ to MFI_PBS_ ratios are close to 1 for CD11b (left panel) and even slightly above 1 for HLA-DR (middle panel) and CD63 (right panel), in all leukocyte subpopulations evaluated. These results demonstrate that the detection of the surface membrane markers is not hampered by hydrogen peroxide, since in this set of experiments (without sputum matrix-derived catalase) cells are exposed to the maximal oxidative power. Therefore, the obtained results definitively discard that the proposed enzymatic liquefaction method has a negative impact on the expression of membrane markers of sputum-resident leukocytes.

**
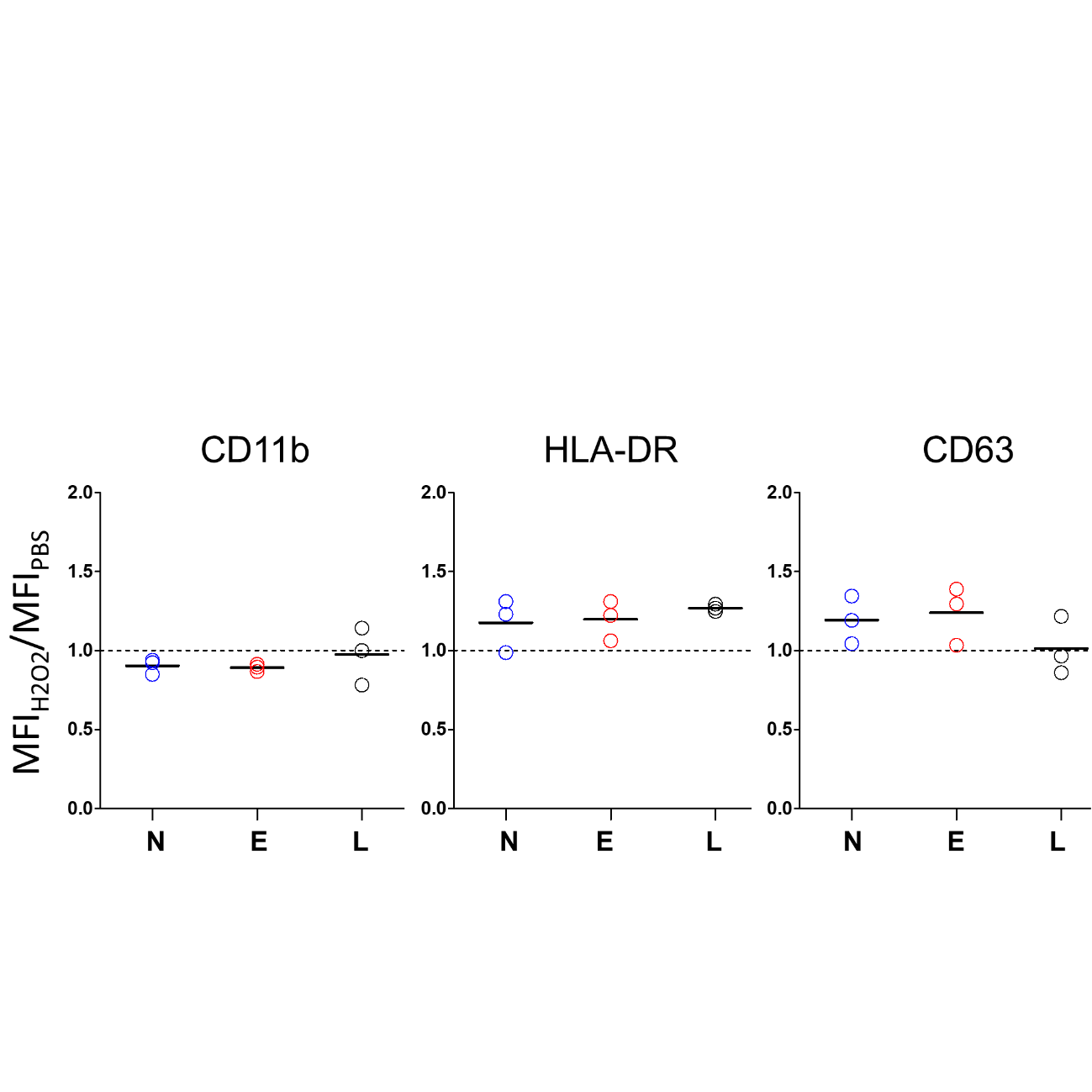
**

**Figure S4. Impact of hydrogen peroxide on CD11b, HLA-DR and CD63 detection in blood leukocytes.** MFI_H2O2_ to MFI_PBS_ ratios of CD11b (left), HLA-DR (middle) and CD63 (right) in neutrophils (N, blue) eosinophils (E, red) and total lymphocytes (L, black) Horizontal bars represent the mean.

**Table S1. Autofluorescence in DAPI-negative leukocytes from liquefied sputum samples.**

| **Cytometer channel**  (Laser,filter, fluorophore) | **Median Fluorescence Intensity on DAPI- cells**  median (IQR) | | | **Kruskall Wallis** |
| --- | --- | --- | --- | --- |
|  | **PBS** (n=11) | **DTT** (n=11) | **Catalase** (n=11) |  |
| Blue, 527/32, FITC | 281.0 (253.0-402.0) | 314.0 (222.0-531.0) | 341.0 (270.0-555.0) | *p*>0.05 |
| Blue, 586/42, PE | 146.0 (129.0-226.0) | 162.0 (125.0-291.0) | 185.0 (144.0-348.0) | *p*>0.05 |
| Blue, 700/54, PCy5 | 27.0 (21.8-65.5) | 28.2 (19.3-61.6) | 34.7 (27.0-71.9) | *p*>0.05 |
| Blue, 783/56, PCy7 | 39.8 (32.1-65.5) | 39.8 (28.2-82.2) | 50.1 (41.1-86.1) | *p*>0.05 |
| Red, 660/10, APC | 89.7 (64.1-92.3) | 87.2 (64.1-92.3) | 76.9 (66.6-87.2) | *p*>0.05 |
| Red, 783/53, APC-Fire | 1.3 (0.0-3.8) | 0.0 (0.0-3.8) | 1.3 (0.0-2.6) | *p*>0.05 |
